# Higher efficacy of direct hemoperfusion using coated activated-charcoal column for disopyramide poisoning: A case report

**DOI:** 10.1097/MD.0000000000008755

**Published:** 2017-12-08

**Authors:** Shigekazu Iguchi, Naotaka Yamaguchi, Hiroki Takami, Takayuki Komatsu, Hirokazu Ookubo, Hajime Sekii, Kenji Inoue, Shinya Okazaki, Iwao Okai, Sonomi Maruyama, Tomohisa Nomura, Manabu Sugita

**Affiliations:** a Department of Emergency and Critical Care Medicine; b Department of Cardiology, Juntendo University Nerima Hospital; c Department of Infectious Diseases, Tokyo Women's Medical University, Tokyo, Japan.

**Keywords:** coated activated-charcoal column, direct hemoperfusion, disopyramide poisoning, half-life (*t*1/2), percutaneous cardiopulmonary support

## Abstract

**Rationale:**

Cases of severe disopyramide poisoning are rare and few have been reported. We report a case in which activated-charcoal column hemoperfusion was dramatically effective for life-threatening disopyramide poisoning.

**Patient concerns:**

A teenage girl who had overdosed on disopyramide (total dose, 4950 mg) was brought to our hospital. She was resuscitated from short period cardiopulmonary arrest and subsequently showed severe cardiogenic shock and ventricular arrhythmia.

**Diagnoses:**

Disopyramide poisoning (self-evident).

Interventions: As hemodynamics remained unstable after providing percutaneous cardiopulmonary support and intra-aortic balloon pumping, we attempted direct hemoperfusion using a coated activated-charcoal hemoperfusion column.

**Outcomes:**

Hemodynamics including electrocardiography and serum disopyramide concentration were dramatically improved, and the patient was ambulatory by hospital day 14.

**Lessons:**

Because disopyramide has low molecular weight and a small distribution volume, blood purification is considered to be the most effective therapy. We selected direct hemoperfusion for relatively high protein-binding rate. In fact, clinical status was dramatically improved, and the calculated half-life of the direct hemoperfusion phase was the shortest of all phases. In cases of severe or life-threatening disopyramide poisoning, blood purification therapy including direct hemoperfusion using a coated activated-charcoal column should be performed.

## 1 Introduction

Disopyramide is a class Ia antiarrhythmic agent used in Japan for the treatment of paroxysmal atrial fibrillation, Wolf–Parkinson–White syndrome, paroxysmal supraventricular tachycardia, and ventricular tachycardia. Recently, the frequency of disopyramide use has reduced, largely due to the influence of the Cardiac Arrhythmia Suppression Trial.^[1,2]^ Disopyramide poisoning has only been reported infrequently, but is very important because of the life-threatening symptoms resulting from relatively small overdoses. In cases of severe disopyramide poisoning, use of activated-charcoal column hemoperfusion is considered effective.^[3]^ We report a case of severe disopyramide poisoning in which use of activated-charcoal column hemoperfusion led to full recovery.

## 2 Case report

**Figure 1:**
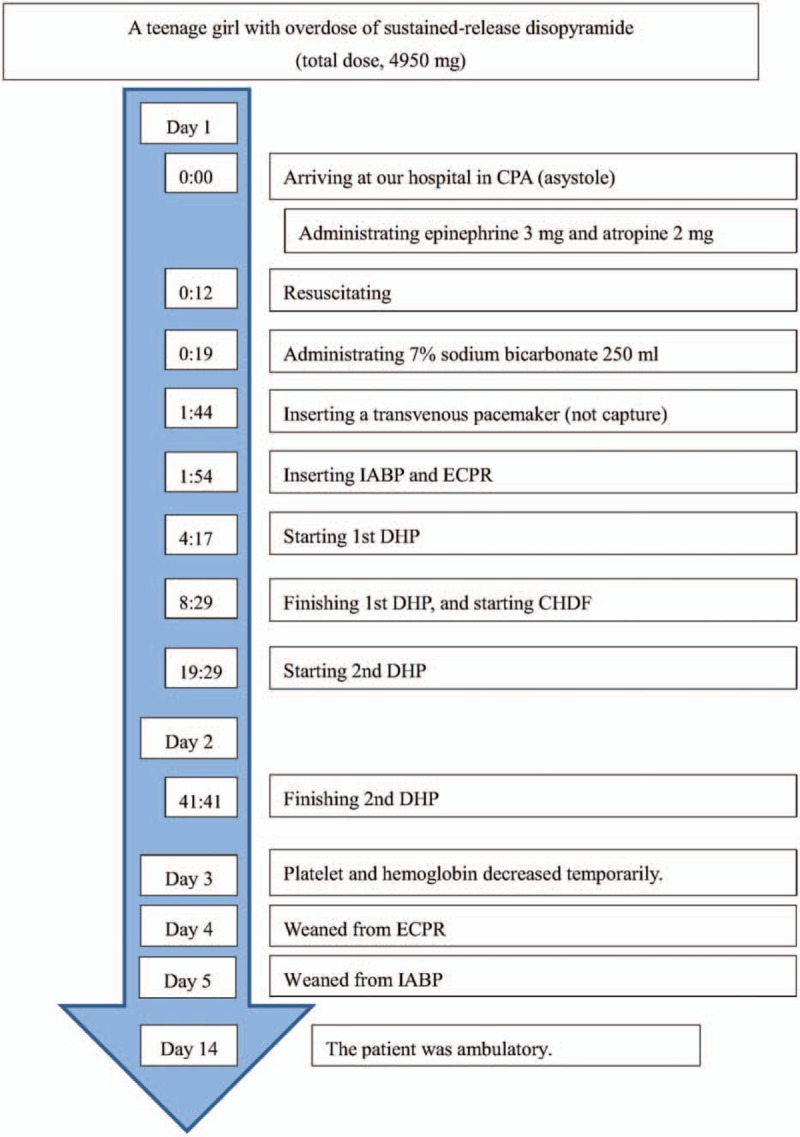
Timeline of this case.

Figure 1 shows timeline of this case.

**Figure 2:**
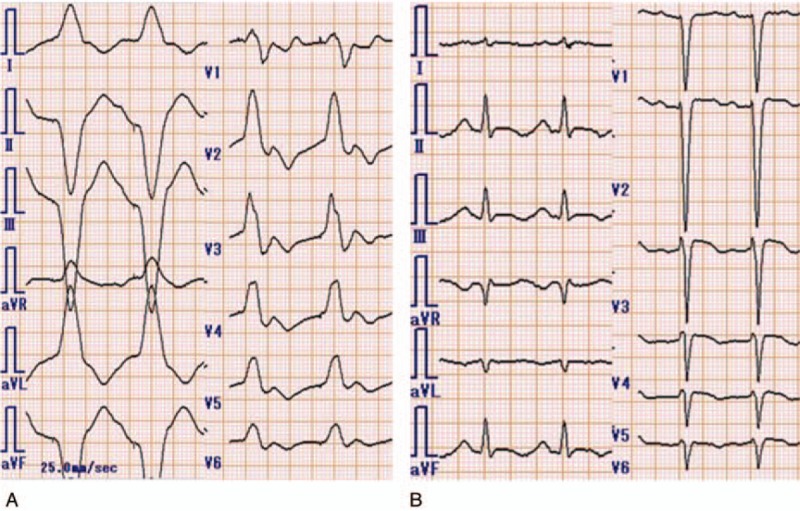
(A) Electrocardiography before direct hemoperfusion (DHP). (B) Electrocardiography after DHP.

**Figure 3:**
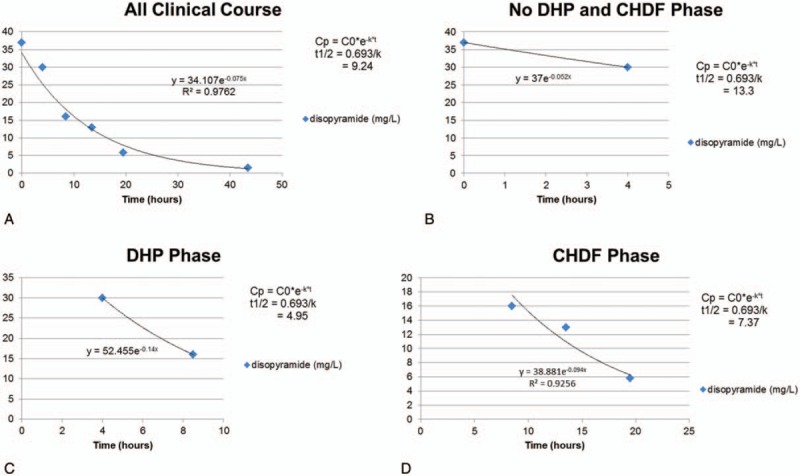
Serum disopyramide concentration transition. We adopt 1-compartment model, and an exponential trendline and differential equation (Cp = C0∗*e*–*k*∗*t*, where Cp = serum drug concentration at *t*, C0 = initial serum drug concentration, *k* = elimination rate constant, *t* = time) were determined. The *t*1/2 was calculated (*t*1/2 = 0.693/*k*). Raw data: 37.0 mg/L at 0 hour, 30.0 mg/L at 4 hours, 16.0 mg/L at 8.5 hours, 13.0 mg/L at 13.5 hours, 5.8 mg/L at 19.5 hours, 1.5 mg/L at 43.5 hours. (A) During all clinical course. (B) Among no continuous hemodiafiltration (CHDF) and direct hemoperfusion (DHP) phase. (C) Among DHP phase. (D) Among CHDF phase.

**Figure 4:**
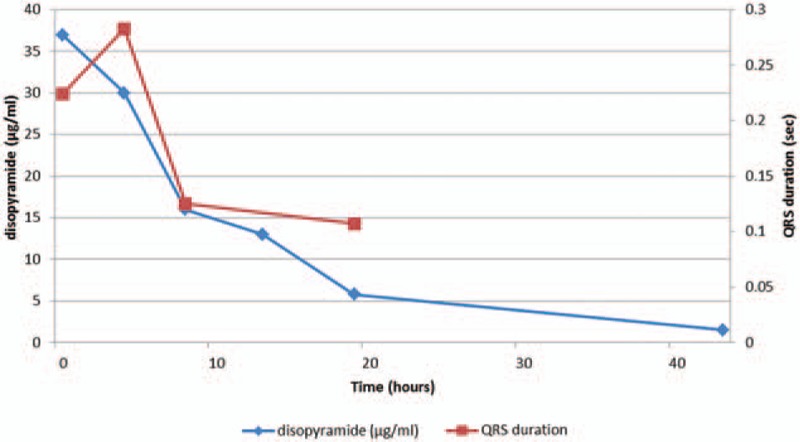
Time series of QRS duration and disopyramide concentration.

A teenage girl with disturbance of consciousness, shock state, and subsequent cardiopulmonary arrest (CPA) was brought to our hospital by ambulance. She had been in an agitated state 30 minutes before the call to emergency medical services. Her family found her unresponsive beside an empty package of sustained-release disopyramide pills (33 × 150-mg Rythmodan R, sustained release product; Sanofi KK, Tokyo, Japan). She entered CPA (asystole) in the ambulance and an emergency medical technician performed cardiopulmonary resuscitation (CPR) based on basic life support. When she arrived at our hospital, she was still in CPA and electrocardiography showed asystole. We continued CPR, intubated the patient, and administrated epinephrine (total, 3 mg) and atropine (total, 2 mg) based on advanced cardiopulmonary life support. As a result, she was resuscitated (at least 16 minutes to return of spontaneous circulation), but remained in a state of severe shock and disturbance of consciousness, showing a wide QRS rhythm with conduction disturbance or non-sustained ventricular tachycardia. As disopyramide poisoning was strongly suggested from the disopyramide packaging and aggressive course, we inserted an oral gastric tube, performed gastric lavage, and administered activated charcoal using the oral gastric tube. At the same time, we administered epinephrine continuously (max 1 μg/kg/min) and 7% sodium bicarbonate 250 mL as a bolus for membrane stabilizing action and correction of severe acidosis. Nevertheless these administrations were not effective because of severe metabolic (lactic) acidosis for no responding cardiogenic shock. Blood gas analysis data were below; before administrating sodium bicarbonate, pH 7.236, HCO_3_- 11.7, Lactate 14.8; after administrating sodium bicarbonate, pH 7.008, HCO_3_- 13.2, Lactate 24.2. As her state remained unchanged, we inserted a transvenous pacemaker. However, we could not capture a rhythm, and performed intra-aortic balloon pumping (IABP) and extracorporeal cardiopulmonary resuscitation (ECPR) in an attempt to stabilize hemodynamics. After minimum stabilizing hemodynamics, we performed direct hemoperfusion (DHP) using a coated activated-charcoal hemoperfusion column (quantity of blood flow: 100 mL/min, Hemosorba CHS-350; Asahi Kasei Medical, Tokyo, Japan). Hemodynamics improved immediately, epinephrine infusion was reduced, and QRS duration on electrocardiography became short and normalized (Fig. 2). After completing DHP therapy, we performed continuous hemodiafiltration (CHDF) as renal replacement therapy, mainly (quantity of blood flow: 100 mL/min, dialysis fluid flow: 500 mL/h, replacement fluid flow: 500 mL/h, filtration fluid flow: 1000 mL/h, column: APF-10S, Asahi Kasei Medical, Tokyo, Japan). Serum concentration of disopyramide decrease from 37.0 mg/L on admission to 5.8 mg/L after 19.5 hours. Figure 3 shows serum disopyramide concentration and calculated half-life (*t*1/2) for the entire course and various treatment situations. Figure 4 shows time series of QRS duration and disopyramide concentration.

As adverse effect of disopyramide, both platelet and hemoglobin decreased on hospital day 3. Platelet count recovered spontaneously and erythrocyte transfusion was needed for anemia. Hypoglycemia was not presented in all clinical courses.

The patient was weaned from ECPR on hospital day 4, and from IABP on hospital day 5, and was ambulatory on hospital day 14.

## 3 Discussion

Since a concentration of disopyramide over 7 mg/L is considered toxic, this case (maximum, 37.0 mg/L) showed very severe poisoning.^[4]^ The indications for hemodialysis (HD) in poisoning care are a low molecular weight (<500 Da), low volume of distribution (<1 L/kg), low degree of protein binding.^[5]^ The molecular weight of disopyramide is 339.47 Da and the distribution volume (Vd) is relatively small (0.8–2 L/kg).^[4]^ We, thus, initially thought that HD may be an effective therapy in this case, but the degree of protein binding is relatively high (20–60%),^[4]^ so we selected DHP using a coated activated-charcoal column at this time. Because the elimination half-life of disopyramide is prolonged in the presence of renal or hepatic dysfunction, we consider DHP as necessary in cases of severe poisoning (such as cases showing shock and organ failure), but not in mild poisoning cases. In a previous article, they concluded DHP was unsuitable for the rapid elimination of disopyramide and β stimulator (isoproterenol) was suitable for stability of hemodynamics.^[6]^ Our patient had severe cardiogenic shock in spite of administrating epinephrine 1 γ (1 μg/kg/min). If hemodynamics was stable, normal hepatic metabolism and renal excretion were expected and hemoperfusion did not need. It was better choice hemoperfusion under instability of hemodynamics despite of enough positive inotropic agents. We think cause of this difference is max disopyramide concentration (more than 2 times).

In our case, DHP phase using the coated activated-charcoal hemoperfusion column is the shortest *t*1/2 (4.95 hours) (Fig. 3). We adopt 1-compartment model, and an exponential trendline and differential equation (Cp = C0∗e^–*k*∗*t*^, where Cp = serum drug concentration at *t*, C0 = initial serum drug concentration, *k* = elimination rate constant, *t* = time) were determined. The *t*1/2 was then calculated (*t*1/2 = 0.693/*k*). Durations for calculating *t*1/2 were established as: entire clinical course; no hemodiafiltration or hemoperfusion phase; direct hemoperfusion phase; and continuous hemodiafiltration phase. The shortest *t*1/2 of all phases was seen in the DHP phase (4.95 hours), followed by the CHDF phase (7.37 hours). The *t*1/2 of the no hemodiafiltration and hemoperfusion phase was 12.4 hours, about 2.5 times that of the DHP phase (Fig. 3). Though we should have quantified the true amount of drug extracted by hemoperfusion, we could not perform for technical problem. Surely, we think that serum concentration of disopyramide transition and calculated half-life are suggested higher efficacy of direct hemoperfusion.

Karim et al^[7]^ reported that HD for extraction of disopyramide offered only one-third the clearance rate of hemoperfusion in vitro. In experimental overdose cases using beagle dogs, over 40% of disopyramide was able to be removed within 3 hours using hemoperfusion.^[8]^ Possibly, HDF may be another better choice. We worried high disopyramide protein binding rate. Disopyramide protein binding rate is 20% to 60% and protein binding rate is relatively low if high serum concentration, generally. However, there is possibility of high protein binding rate because of high alpha-acid glycoprotein (AAG) concentration. AAG is elevated under renal dysfunction, inflammation, and others. Hirata et al^[9]^ reported disopyramide protein binding rate of a continuous ambulatory peritoneal dialysis patient was 85%. As our patient had severe cardiogenic shock and acute renal dysfunction, elevation of AAG concentration and disopyramide protein binding rate was assumed and it is possible hemoperfusion was more efficient.

Recently, cases of severe disopyramide poisoning have been reported in which blood purification therapy including DHP was not performed or mentioned. This may be understandable in cases involving hyper-acute fatal or chronic poisoning,^[10,11]^ but the perceived importance of blood purification therapy including DHP in disopyramide poisoning treatment may be fading. We, thus, wish to emphasize that in cases of severe or life-threatening disopyramide poisoning, blood purification therapy including DHP using coated activated charcoal column therapy appears warranted.

### 3.1 Ethics approval and consent to participate

We got the patient's consent for the publication of this case report. This report does not contain any information that could be used to identify the patient and we received approval of the Ethics Review Board of our hospital (Approval No. 15–52).
